# Development of the dementia attitude scale focusing on well-being

**DOI:** 10.3389/fpsyg.2024.1410048

**Published:** 2024-11-28

**Authors:** Ichie Ono, Hisae Nakatani, Yuriko Inoue, Xuxin Peng, Hironobu Hamada

**Affiliations:** ^1^Department of Community and Public Health Nursing, Graduate School of Biomedical and Health Sciences, Hiroshima University, Hiroshima, Japan; ^2^Department of Physical Analysis and Therapeutic Sciences, Graduate School of Biomedical and Health Sciences, Hiroshima University, Hiroshima, Japan

**Keywords:** dementia, attitude, scale development, well-being, Japan

## Abstract

**Introduction:**

This study aimed to develop the Dementia Attitude Scale Focusing on well-being (DASFWB) and to verify its reliability and validity. This scale measures the factors that individuals without dementia would consider important for their well-being if they were to develop dementia. It is expected to serve as a useful indicator for intervention strategies aimed at achieving an inclusive society.

**Methods:**

The draft DASFWB scale was developed by extracting stories from older adults with mild Alzheimer's disease. The questionnaire was distributed to 1,614 adults aged 65 and older who were able to complete the self-administered questionnaire. Data from 815 individuals who completed the questionnaire without help (58.8% valid response rate) were analyzed. Exploratory and confirmatory factor analyses were performed to identify the factors underlying the scale. Reliability was tested using Cronbach's alpha. Validity was tested through sample, criterion-related, convergent, and discriminant validity.

**Results:**

The development and validation of the DASFWB identified a three-factor, 12-item scale. Cronbach's alpha coefficients for the scale and its three factors were 0.857, 0.843, 0.723, and 0.644, respectively. The confirmatory factor analysis model indices were as follows: χ^2^ = 146.574, *df* = 51, *p* < 0.001, comparative goodness of fit index = 0.930, goodness of fit index = 0.945, Tucker-Lewis index = 0.909, and root mean square error of approximation = 0.068. The composite reliability value for convergent validity, which was >0.7, was higher than the average variance extracted value. The criterion-related validity showed a weak correlation (ρ = −0.245 to 0.341, *p* < 0.001).

**Discussion:**

The DASFWB exhibits good reliability and validity, indicating its utility as a measuring instrument.

## 1 Introduction

The prevalence of dementia increases with age (Lopez and Kuller, 2019), and the estimated number of people with dementia worldwide was 55.2 million in 2019, rising to 78 million in 2030 and 139 million in 2050 [World Health Organization (WHO, 2021a)]. In this context, stigma and discrimination affecting people with dementia are widespread worldwide and represent significant challenges that need to be addressed (WHO, 2021b). According to Goffman (1963), stigma is defined as “an attribute that is deeply discrediting,” which reduces the bearer “from a whole and usual person to a tainted, discounted one.” Stigma related to mental illness, including dementia, is a negative and inaccurate attitude toward a target group, characterized by stereotyping (cognitive aspect), prejudice (attitudinal aspect), and discrimination (behavioral aspect) (Corrigan and Penn, 2015; Matsumoto et al., 2024). Among these aspects, the attitudinal aspect is the central construct of stigma and has been most frequently employed as an outcome of dementia-friendly programs (Matsumoto et al., 2023).

Additionally, stigma can be categorized into self-stigma, public stigma, and courtesy stigma (Alzheimer's Disease International, 2019). Individuals with dementia experience self-stigma, characterized by the internalization of negative attitudes and behaviors associated with dementia. Public stigma involves negative attitudes and behaviors directed toward people with dementia. Courtesy stigma refers to the negative behaviors directed toward the family, friends, or close associates of individuals with dementia.

To reduce stigma toward people with dementia, WHO (2021a) set a goal for 75% of member countries to develop a national policy on dementia by 2025. In Japan, the Ministry of Health, Labour and Welfare initiated a comprehensive effort to establish dementia policies in 2013 (Nakanishi and Nakashima, 2014). In June 2023, the Basic Act on Dementia to Promote an Inclusive Society was promulgated. The act aims to promote a vibrant society in which each person, including those with dementia, can fully realize their individuality and abilities and live in an inclusive and mutually-supportive social environment with others while respecting each person's character and individuality (Japanese Law Translation, 2023).

Previous research has pointed out that the existing scales for evaluating stigma and attitudes related to dementia have limitations regarding their target populations and applications (O'Connor and McFadden, 2010; Piver et al., 2013) and are not sufficiently established (Herrmann et al., 2018). Noguchi et al. (2022) developed a scale that assesses multidimensional public stigma, focusing on a participant's response to a hypothetical dementia diagnosis as well as people's attitudes toward a person with dementia. Matsumoto et al. (2024) developed a shortened version of the scale, originally developed by Kim and Kuroda (2011), to measure both positive and negative attitudes toward people with dementia. This version was developed to facilitate large-scale community and local government surveys. Furthermore, Bhatt et al. (2023) developed the Discrimination and Stigma Scale Ultra Short for People Living with Dementia to enable WHO member countries to assess stigma toward people with dementia at a regional level.

Stigma can worsen the mental health of people with dementia (WHO, 2021b). Previous research has demonstrated that stigma toward individuals with dementia is negatively correlated with well-being (Noguchi et al., 2022). well-being refers to a positive psychological state in which individuals feel good about themselves, serving as an important indicator of mental health (Ryan and Deci, 2001). This topic has garnered significant international interest, with numerous studies on well-being conducted in various countries (Huang et al., 2022). It has been noted that the well-being of individuals with dementia can deteriorate due to a lack of “continuity of experience,” which is related to memory and identity (Clare et al., 2020). However, while some individuals are able to cope positively with their dementia, others may suffer from fear and anxiety (Xanthopoulou and McCabe, 2019; Huizenga et al., 2022). This difference is believed to be significantly influenced by understanding of dementia and societal attitudes toward it (Eriksen et al., 2016). Therefore, eliminating discrimination and prejudice against individuals with dementia is crucial for enhancing well-being (Phinney et al., 2023).

This study aims to develop the Dementia Attitude Scale Focusing on well-being (DASFWB) and to verify its reliability and validity. This scale measures the factors that people without dementia would consider important for well-being if they were to develop dementia. While scales have been developed to evaluate stigma and attitudes toward dementia and people with dementia in pursuit of an inclusive society (Kim et al., 2021; Noguchi et al., 2022; Bhatt et al., 2023), none of them focus on well-being in every item. We designed a scale to ask questions framed with the positive phrase “even if one develops dementia,” allowing individuals to reflect on attitudes that are important for well-being from the perspective of those affected. This scale is expected to serve as a useful indicator for intervention strategies aimed at achieving an inclusive society.

## 2 Definition of terms

In this study, based on the definition by Kim and Kuroda (2011), we define attitudes toward dementia as encompassing both positive and negative feelings, as well as accepting and rejecting behaviors.

## 3 Methods

### 3.1 Study design

This study employed a cross-sectional design and a self-administered questionnaire without identifiable markings.

### 3.2 Creation of the draft scale

#### 3.2.1 Conceptual structure and scale item creation

The scale was developed based on a semi-structured interview survey of seven older adults diagnosed with mild Alzheimer's disease (Ono and Nakatani, 2021). Two interviews were conducted: the first within 1 year of diagnosis and the second 1 year after the first interview. Having a role in life, a place to engage and participate with others, family and friends to confide in, support to accepting the diagnosis, and optimism for the future are all necessary for older adults with dementia to have a better life (Eriksen et al., 2016; Bronner et al., 2016; JDWG, 2018). With reference to previous studies, the interviews comprised discussions regarding (1) the individual's position within the household, participation in community activities, and daily struggles after being diagnosed with dementia; (2) their acceptance of the diagnosis; and (3) their hopes for future support with daily living. Focusing solely on the interview context, the study categorized 23 items and derived four categories: “distress toward knowing they have dementia,” “emotion of hope and despair toward their family,” “anxiety about socializing,” and “desire to live authentically in a familiar community.” Many items were related to the distress of having dementia and difficulties in daily life caused by the symptoms, which diminished the well-being of older adults. We also found that the participants maintained their well-being and wanted to live while interacting with people in the community, including persons with dementia. A draft of the DASFWB was developed based on the categories generated from the interview narratives.

#### 3.2.2 Verification of content validity

To assess the validity of the 23-item scale, five certified nurses in dementia (Dementia Certified Nurses: DCN) and a university faculty member with dementia research experience were asked for their opinions on item modifications. DCNs are nursing professionals with advanced training and certification in dementia nursing. They provide advanced care to support patients with dementia and their families. Additionally, they offer guidance and consultation to other nursing professionals, leveraging their specialized knowledge and skills in dementia nursing. They also collaborate with other nursing and medical professionals to deliver care that respects the life, quality of life (QOL), and dignity of patients with dementia (Taneichi and Rokkaku, 2019). The DCN was asked to comment on: (1) whether the items were correctly worded; (2) whether they reflected well-being from the perspective of a person with dementia; (3) whether the items were similar in content; (4) whether the items were eligible to remain on the scale; and (5) which items should be added. Based on their feedback, five items were combined and two items were added. The Content Validity Index (CVI) was used to assess the content validity of the remaining 17 items; a CVI of 0.79 or higher indicates high content validity (Polit and Beck, 2006). Five DCNs rated each scale item on a 4-point scale from “adequate” to “not adequate.” As a result, all 17 items were adopted with a CVI of 0.80 or higher. Afterwards, the items were reviewed to see if they were easy for older adults to understand and not psychologically stressful and, finally, a CVI of 1 was achieved.

#### 3.2.3 Validation of face validity

Face validity was tested in a pilot study with 10 older adults. The participants completed a questionnaire regarding whether the item meanings were clear and understandable, whether there was any ambiguity in the responses, and whether they experienced any difficulties when providing responses. No problems were identified related to ease of comprehension or burden in providing responses to the 17 items.

### 3.3 Participants and sample size

The participants were older adults living in a town with a population of 27,000, located in a rural area with a 40% aging rate. They were members of a community-based organization supported by national and local governments to promote enjoyable activities for older adults (Japan Federation of Senior Citizens Clubs, n.d.). The reason for selecting a single municipality was to avoid bias in public support for older adults. Participants were divided into two groups for exploratory factor analysis (EFA) and confirmatory factor analysis (CFA). EFA required at least 10 participants per item (Carneiro, 2003) and CFA 200 participants (Anderson and Gerbing, 1984). Therefore, over 370 participants were required for EFA and CFA in the current study. The response rate we expected for the questionnaires was ~45% based on previous studies (Ozone et al., 2022). Furthermore, as some older adults who use long-term care insurance have cognitive decline (Ministry of Health, 2002), we excluded older adults with this type of insurance from the analysis, as their responses may not have been valid. In Japan, the certification rate for long-term care insurance is 18.4% (Cabinet Office, 2022). Based on the above, the questionnaire needed to be distributed to over 995 older adults; it was ultimately distributed to 1,614 older adults.

Following distribution, the questionnaires were collected by the club's senior officers. Participants were asked to hand in their sealed questionnaires to a senior club officer. Data were collected from November 2022 to January 2023.

### 3.4 Measures

The survey items included questions on basic characteristics, original draft of the DASFWB, and three different scales used to assess criterion-related validity.

#### 3.4.1 Basic characteristics

Basic characteristics included questions on age, sex, marital status, sense of economic insecurity, living with family, use of long-term care insurance, presence of illness, and subjective symptoms of dementia (Ura et al., 2015; Miyamae et al., 2016; Kawamura et al., 2023).

#### 3.4.2 Original draft of the DASFWB

The DASFWB was rated on a five-point scale as follows: 5 = agree, 4 = somewhat agree, 3 = undecided, 2 = not so much agree, and 1 = disagree. Higher scores indicated higher levels of well-being.

#### 3.4.3 Criterion-related validity

The criterion-related validity measures included the 11-item revised Philadelphia Geriatric Center Morale Scale (PGC-MS) (Liang et al., 1992), the Japanese Lubben Social Network Scale (LSNS-6) short version (Kurimoto et al., 2011), and the revised Japanese version (Arimoto and Tadaka, 2019) of the University of California, Los Angeles Loneliness (UCLA-Loneliness) Scale.

#### 3.4.4 The PGC-MS

The PGC-MS was developed and validated as a measure of subjective well-being among older adults and comprises three factors: psychological agitation, attitudes toward aging, and feelings of loneliness and dissatisfaction. The 11-item scale's short version total score scale ranged from 0 to 11, with one point for positive responses and zero for other responses; higher total scores indicated higher subjective well-being. This scale was chosen because it is believed that the psychological distress caused by stigma may affect well-being; it is expected to have a positive correlation with the DASFWB.

#### 3.4.5 The LSNS-6

The reliability and validity of the LSNS-6 have been demonstrated (Lubben et al., 2006). This scale is implemented to determine the size of an individual's family, community, and network of friends. A higher score indicates a larger social network; indeed, social interactions are associated with quality of life and cognitive decline in older adults (Moreno et al., 2020; Evans et al., 2018). This scale considers reducing social networks due to stigma as an important factor for well-being, and it is assumed to have a positive correlation with the DASFWB.

#### 3.4.6 The UCLA-loneliness

The UCLA-Loneliness scale quantifies an individual's loneliness level rather than measuring the extent of their social network. Higher scores on this scale indicate increased levels of perceived loneliness. Its reliability and validity have been empirically established (Saito et al., 2019). Loneliness has been linked to an increase in internalized stigma, leading individuals to accept societal labels, alienate themselves from others, and thus experience greater loneliness (Yildirim and Kavak Budak, 2020). Therefore, the choice of this scale is based on the hypothesis that it has a negative correlation with the DASFWB.

### 3.5 Data analysis

IBM SPSS Statistics standard Grand Pack 28.0 and IBM SPSS Amos version 29.0 were used for the data analysis.

#### 3.5.1 Item analysis

To test the reliability and ceiling and floor effects, inter-item and item–total (I–T) correlations were examined. The ceiling effect was set to mean +5 standard deviation points, and the floor effect was set to mean −1 standard deviation point. As highly correlated coefficients may influence the results, inter-item correlation was set to *r* > 0.75 (Yusoff et al., 2021). For the I–T correlation, *r* < 0.3 was set as the criterion for exclusion (Yusoff et al., 2021).

#### 3.5.2 Reliability study

Cronbach's alpha coefficients were calculated for both the scale as a whole and each factor, to assess reliability. The criterion for the coefficient was set to > 0.7 (Yusoff et al., 2021).

#### 3.5.3 EFA

The Kaiser-Meyer-Olkin (KMO) test and Bartlett's sphericity test were performed to determine the appropriateness of factor analysis. The typical range for KMO values is between 0.8 and 1.0, while the value produced by Bartlett's sphericity test is considered significant if it is < 0.05 (Williams et al., 2010). Factor analysis was performed using maximum likelihood and Promax rotation. We removed commonalities below 0.2 and factor loadings under 0.3 (Boateng et al., 2018). Subsequently, we verified the factor structure and assigned descriptive titles to each factor.

#### 3.5.4 CFA

CFA was performed to test factor structure and the model's goodness of fit. The goodness of fit index, comparative fit index, and Tucker-Lewis index are within a range of 0–1. A good fit is generally declared if the value is >0.9, while root mean square error of approximation is seen as a good fit if the value is < 0.08 (Yusoff et al., 2021).

#### 3.5.5 Convergent and discriminant validity

Composite reliability (CR) and average variance extracted (AVE) were used to assess convergent validity. A scale is considered to have good convergent validity when AVE > 0.5 and CR > 0.7; when 0.36 < AVE < 0.5, the scale is considered to have acceptable convergent validity (Shrestha, 2021). Discriminant validity is assessed by comparing the square root of the AVE with the correlation coefficient between factors. A scale has good discriminant validity if the correlation coefficient between factors is less than the corresponding square root of the AVE (Sahoo, 2019).

#### 3.5.6 Criterion-related validity

To ensure criterion-related validity, the relationships between the developed scale, PGC-MS, LSNS-6, and UCLA-Loneliness were confirmed using Spearman's rank correlation coefficient.

### 3.6 Ethical considerations

This study involving human participants was approved by the ethics committee of epidemiology research at Hiroshima University (approval number E2022-0020). Participants were informed of the purpose and methods of the study and told that they were free to participate or withdraw from the study at any point without penalty or consequences. Furthermore, individuals would be unable to be identified based on their data. The participants provided their written informed consent to participate in this study. This study was conducted in accordance with the ethical standards established by the Declaration of Helsinki.

## 4 Results

### 4.1 Participant characteristics

Of the 1,614 questionnaires distributed, responses from 815 individuals were analyzed (valid response rate: 50.5%). Participant characteristics are presented in Table 1. This study included 382 male (46.9%) and 433 female (53.1%) participants. The age group with the highest number of participants was 75–85 years, comprising 453 individuals (55.6%). Of the participants, 150 (18.5%) were living alone, 663 (81.5%) were living together, 668 (82.2%) reported having an illness under treatment, and 160 (19.6%) had subjective symptoms of dementia. No differences were found between the 408 EFA and 407 CFA participants.

**Table 1:** Participant characteristics.

**Item**	**Description**	**Total (*****n*** = **815)**		**EFA (*****n*** = **408)**		**CFA (*****n*** = **407)**		***P***
		***n***	**%**	***n***	**%**	***n***	**%**	
Sex	Male	382	46.9	184	45.1	198	48.6	0.310
	Female	433	53.1	224	54.9	209	51.4	
Age	65–74 years	182	22.3	87	21.3	95	23.3	0.557
	75–84 years	453	55.6	225	55.1	228	56.0	
	≥85 years	180	22.1	96	23.5	84	20.6	
Marital status	Married	809	99.4	405	99.5	404	99.3	0.654
	Unmarried	5	0.6	2	0.5	3	0.7	
Household composition	Living alone	150	18.5	76	18.4	76	18.5	0.987
	Living together	663	81.5	332	81.6	331	81.5	
Sense economic insecurity	Not worried	384	47.6	200	49.8	45.4	45.4	0.216
	Worried	421	52.4	202	50.2	54.6	54.6	
Illness	No	668	82.2	336	82.3	334	82.0	0.914
	Yes	145	17.8	72	17.7	73	18.0	
Subjective symptoms of dementia	No	665	80.4	330	81.1	325	79.7	
	Yes	160	19.6	77	18.9	83	20.3	0.609

P, chi-square test; CFA, confirmatory factor analysis; EFA, exploratory factor analysis.

### 4.2 Item analysis

In this study, three out of the 17 total items exhibited ceiling effects (item 3: 4.11 ± 1.016, item 16: 4.450 ± 0.819, item 17: 4.020 ± 0.984). No floor effect items were present. The ceiling effect item was retained after careful consideration of its content by the experts. No item exhibited *r* > 0.75 in the inter-item correlations, and the I–T correlations with *r* < 0.30 (item 4: *r* = 0.249, item 11: *r* = 0.043) were deleted (Table 2).

**Table 2:** Initial item analysis of the Dementia Attitude Scale Focusing on well-being.

	**Item**	**Mean value**	**Standard deviation**	**Ceiling effect**	**Floor effect**	**Item–total correlation**
1	I won't feel depressed if I have dementia.	3.400	1.002	4.402	2.398	0.479^**^
2	I believe I'm still valuable even if I have dementia.	3.610	1.103	4.713	2.507	0.619^**^
3	I'd like to receive treatment and advice if I have dementia.	4.110	1.016	5.126	3.094	0.410^**^
4	I believe my mood will suffer from harsh words and attitudes from family members when I have dementia.	2.800	1.070	3.870	1.730	0.249^**†^
5	I believe my opinion will still be respected if I have dementia.	3.240	0.927	4.167	2.313	0.642^**^
6	I think my family will assist me even if I forget things due to dementia.	3.940	0.973	4.913	2.967	0.560^**^
7	I don't think I'd feel ashamed even when I make mistakes due to loss of memory (dementia).	3.140	1.084	4.224	2.056	0.518^**^
8	I think I can live with dementia without worrying about what people around me will think.	3.080	0.998	4.078	2.082	0.659^**^
9	I'd prefer living in my home while receiving nursing services even if I develop dementia.	3.920	1.040	4.960	2.880	0.537^**^
10	I think that if I have dementia, I will be able to accept myself with dementia.	3.530	0.911	4.441	2.619	0.627^**^
11	I'm worried that if I get dementia, I will be a nuisance to those around me.	1.980	0.874	2.854	1.106	0.043^†^
12	I think people in my community and friends will support me despite my dementia.	3.180	0.959	4.139	2.221	0.692^**^
13	I would like to keep in touch with my community and friends even if I have dementia.	3.780	1.043	4.823	2.737	0.694^**^
14	I believe that I will have a role in society despite having dementia.	3.340	1.143	4.483	2.197	0.720^**^
15	I think I can still live the life I desire despite having dementia.	3.090	1.070	4.160	2.020	0.774^**^
16	I want to try to avoid getting dementia.	4.450	0.819	5.269	3.631	0.435^**^
17	I have someone I can talk to when I suspect dementia.	4.020	0.984	5.004	3.036	0.550^**^

^**^P < 0.001.

^†^Item-total correlations of *r* < 0.30 were excluded.

### 4.3 EFA

EFA was performed using maximum likelihood and Promax rotation, while the KMO and Bartlett's sphericity tests were conducted prior to EFA to determine the factor analysis' goodness of fit; the KMO value was 0.885, and Bartlett's sphericity test was *p* < 0.001, while EFA implementation was reasonable. The number of factors was extracted by adopting a three-factor structure that exhibited more than 1 in the eigenvalue analysis of the correlation matrix. Items with commonality < 0.20 or less, and factor loadings < 0.30, were deleted, resulting in a three-factor, 12-item scale (Table 3). The items deleted were item 1, “I won't feel depressed if I have dementia,” item 2, “I believe I'm still valuable even if I have dementia,” and item 6, “I think my family will assist me even if I forget things due to dementia.”

**Table 3:** Exploratory factor analysis of the Dementia Attitude Scale Focusing on well-being.

	**Item**	**Factor 1**	**Factor 2**	**Factor 3**	**Cronbach's α coefficient**
Factor 1	Willingness to engage in society				0.843
15	I think I can still live the life I desire despite having dementia.	0.861	0.057	−0.129	
14	I believe that I will have a role in society despite having dementia.	0.804	−0.084	0.064	
12	I think people in my community and friends will support me despite my dementia.	0.723	0.012	−0.030	
13	I would like to keep in touch with my community and friends even if I have dementia.	0.704	−0.045	0.097	
5	I believe my opinion will still be respected if I have dementia.	0.388	0.211	0.045	
Factor 2	Dispelling anxiety about dementia				0.723
8	I think I can live with dementia without worrying about what people around me will think.	0.071	0.828	−0.092	
7	I don't think I'd feel ashamed even when I make mistakes due to loss of memory (dementia).	−0.134	0.749	0.037	
10	I think that if I have dementia, I will be able to accept myself with dementia.	0.216	0.416	0.068	
9	I'd prefer living in my home while receiving nursing services even if I develop dementia.	0.137	0.317	0.138	
Factor 3	Actions to take to cope with dementia				0.644
16	I want to try to avoid getting dementia.	−0.076	−0.013	0.799	
3	I'd like to receive treatment and advice if I have dementia.	0.006	0.038	0.516	
17	I have someone I can talk to when I suspect dementia.	0.230	0.018	0.474	
	Cronbach α for the overall scale.				0.857
	Eigenvalues	4.790	1.416	1.013	
	Factorial correlation				
	Total	0.889	0.762	0.657	
	Factor 1	1.000	0.526	0.457	
	Factor 2		1.000	0.310	
	Factor 3			1.000	

Factor extraction: maximum likelihood. Rotation: Promax rotation.

Factor 1 comprised five items denoting “willingness to engage with society,” as it revealed a distinct way of living and interacting with the community after dementia onset. Factor 2 comprised four items and was labeled “dispelling anxiety about dementia,” owing to its emphasis on accepting the disease and avoiding concerns about failure. Factor 3 comprised three items; it was named “actions to take to cope with dementia,” since it indicated efforts to prevent dementia and a willingness to promote health, such as seeking treatment and consultation, when symptoms of dementia were perceived. The Cronbach's alpha coefficients for the scale and individual factors were 0.843 for Factor 1, 0.723 for Factor 2, 0.644 for Factor 3, and 0.857 for the overall scale. The coefficient correlations ranged from ρ = 0.310–0.526, indicating a moderate to weak correlation.

### 4.4 CFA

**Figure 1:**
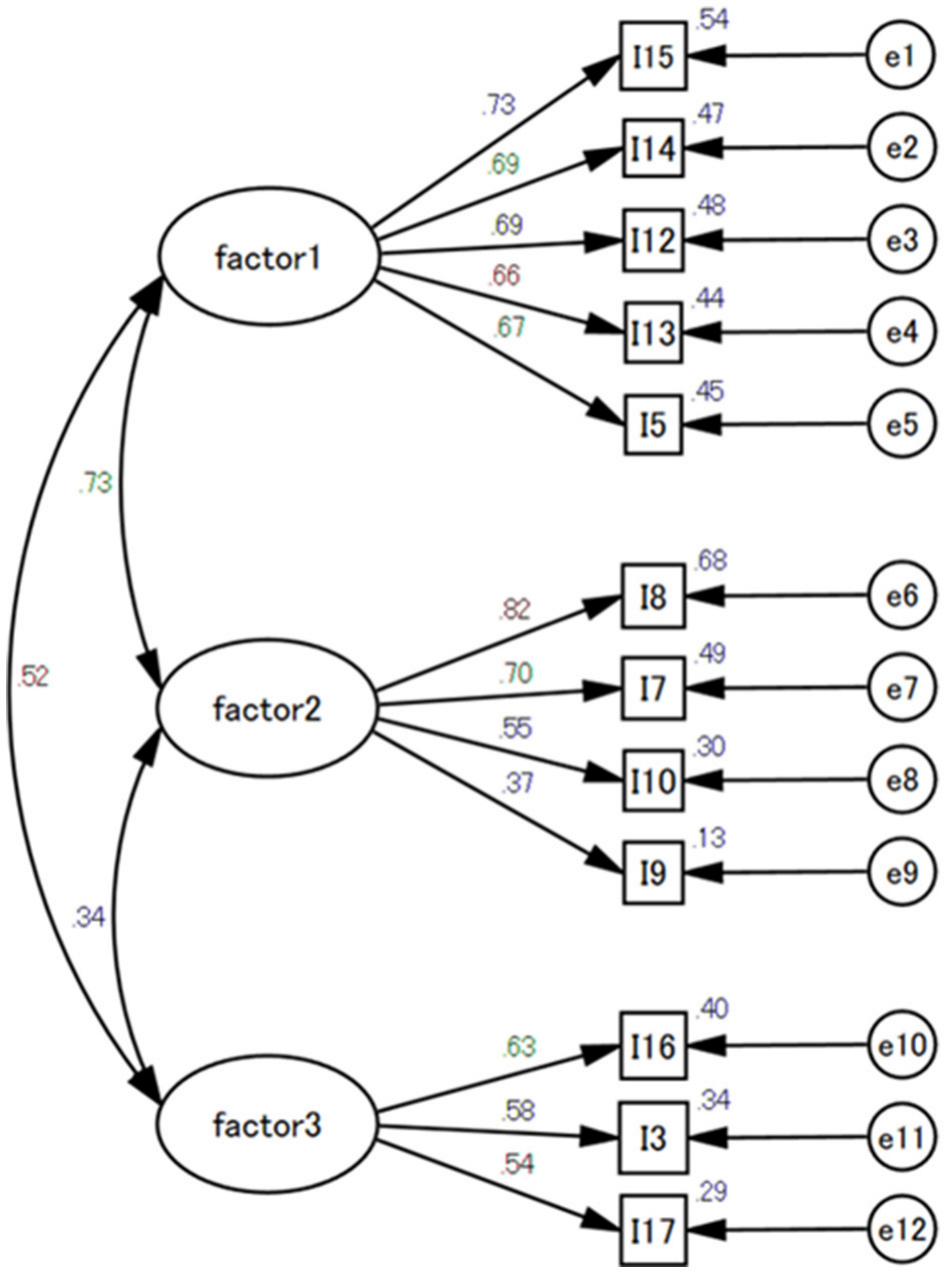
Confirmatory factor analysis of the Dementia Attitude Scale Focusing on well-being. CFI, comparative fit index; GFI, goodness fit index; TLI, Tucker-Lewis index; RMSEA, root mean square error of approximation. X^2^= 146.574; df= 51; CFI = 0.930; GFI= 0.945; TLI= 0.909; RMSEA = 0.068.

CFA was conducted to test the goodness of fit of the three-factor structures obtained in the EFA. The results indicate that the 12 items of the three factors demonstrated a favorable goodness of fit, with χ^2^ = 146.574, *df* = 51, *p* < 0.001, Tucker-Lewis index = 0.909, comparative of fit index = 0.930, goodness of fit index = 0.945, and root mean square error of approximation = 0.068 (Figure 1).

### 4.5 Convergent and discriminant validity

Convergent validity was assessed by analyzing the AVE and CR of the three factors. The AVE values for Factors 1, 2, and 3 were 0.49, 0.46, and 0.53, respectively, while the CR values were 0.82, 0.76, and 0.77, respectively. For Factors 1 and 2, the AVE ranged from 0.36 to 0.5, which was slightly below the criterion of 0.5. However, CR was over 0.7 for all factors (Table 4).

**Table 4:** Criterion-related, convergent, and discriminant validity.

	**DASFWB**			
	**Overall**	**Factor 1**	**Factor 2**	**Factor 3**
PGC-MS	0.341^**^	0.290^**^	0.260^**^	0.232^**^
LSNS-6	0.191^**^	0.183^**^	0.055	0.244^**^
UCLA-Loneliness	−0.245^**^	−0.250^**^	−0.127^*^	−0.168^**^
√AVE		0.70	0.68	0.73
AVE		0.49	0.46	0.53
CR		0.82	0.76	0.77

DASFWB, Dementia Attitude Scale Focusing on Well-being; PGC-MS, 11-item revised Philadelphia Geriatric Center Morale Scale; LSNS-6, Japanese Lubben Social Network Scale; UCLA-Loneliness, UCLA Loneliness scale; √AVE, root average variance extracted; AVE, average variance extracted; CR, composite reliability. Criterion-related validity: PGC-MS, LSNS-6, UCLA. Convergent and discriminant validity: √AVE, AVE, CR. Spearman's rank correlation coefficient: ^**^p < 0.001, ^*^p < 0.05.

Regarding discriminant validity, the square root of the AVE for all factors was greater than their corresponding correlation coefficients (Table 4).

### 4.6 Criterion-related validity

To evaluate the criterion-related validity, we calculated the correlation coefficients between the newly created scale and the PGC-MS, LSNS-6, and UCLA-Loneliness (Table 4). The correlation with PGC-MS was weak for the entire scale, ρ = 0.341 (*p* < 0.001), and for Factors 1–3, ranging from ρ = 0.232 to 0.290 (*p* < 0.001). For the LSNS-6, the overall score was ρ = 0.191 (*p* < 0.001), with Factors 1 and 3 having correlation coefficients of ρ = 0.183 (*p* < 0.001) and ρ = 0.244 (*p* < 0.001), respectively; Factor 2, however, was uncorrelated, with a coefficient of ρ = 0.055. UCLA-Loneliness had correlation coefficients of ρ = −0.245 (*p* < 0.001) for the entire scale, ρ = −0.250 (*p* < 0.001) for Factor 1, ρ = −0.127 (*p* < 0.05) for Factor 2, and ρ = −0.168 (*p* < 0.001) for Factor 3.

## 5 Discussion

### 5.1 Contents of the scale

The feelings of older adults with dementia used in the draft scale comprised four categories, whereas the DASFWB consisted of three factors and 12 items identified through the EFA. The DASFWB consists of three factors: “willingness to engage in society,” “dispelling anxiety about dementia,” and “actions to take to cope with dementia.” All items assume that a person without dementia has developed it. In addition, the focus on well-being is expected to foster positive attitudes toward dementia and help develop an inclusive society.

Factor 1, “willingness to engage in society,” which comprised one category of the conceptual construct, namely the “desire to live authentically in a familiar community,” and one item, the “emotion of hope and despair toward their family.” This factor represents an aspiration to reside in the community and interact with other community members. The “willingness to engage in society” factor in this scale reflects the view that social interactions play an important role in well-being. A sense of connection and belonging in a society where people's dignity is respected and are encouraged to engage in social networks is essential for their well-being and quality of life (Wiersma and Denton, 2016). However, the stigma associated with dementia can lead to social exclusion, abuse, and discrimination (WHO, 2021b), negatively affecting the well-being of people with dementia. Therefore, maintaining connections with the society is considered an important factor for well-being.

Factor 2, “dispelling anxiety about dementia,” includes an item in the construct category “anxiety about socializing.” This item is related to psychological well-being, such as being able to live one's life without worrying about what others think of one's dementia and being able to accept the illness. Despite the ongoing global efforts aimed at eliminating stigma toward people with dementia, inadequate awareness of dementia continues to subject those with dementia and their caregivers and families to prejudice and discrimination in their communities (WHO, 2021b). The stigma associated with dementia is negatively correlated with quality of life (Lion et al., 2020) and can lead to psychological distress, such as depression and anxiety (Sibley et al., 2021). Such psychological burdens can lead to a decline in well-being; “Dispelling anxiety about dementia” becomes an important factor for the well-being of individuals who hypothetically develop the condition.

Factor 3, “actions to take to cope with dementia,” assesses whether respondents can take actions on their own to prevent dementia and adopt countermeasures when they observe a decline in cognitive function. As everyone is at risk of developing dementia, it is vital to take measures to prevent and manage cognitive decline (Lisko et al., 2021). However, according to the report, ~20% of people with dementia wish to keep their diagnosis a secret (Alzheimer's Disease International, 2019). Shame may serve as an underlying mechanism through which stigma is enacted and perpetuated, resulting in delays in accessing diagnosis and support services (Lopez et al., 2020). The willingness to seek medical advice or consultation without hesitation when suspecting the onset of dementia is an important factor for well-being.

### 5.2 The reliability and validity of the scale

The reliability and validity of the DASFWB were tested using items, exploratory and confirmatory factors, constructs, and criterion-related analyses. After analyzing the items and conducting EFA, we identified a structure comprising 12 items and three factors from the draft of 17 items. Cronbach's alpha coefficients exceeded 0.7 overall for Factor 1, “willingness to engage in society,” and Factor 2, “dispelling anxiety about dementia.” However, Factor 3, “actions to take to cope with dementia,” yielded a lower alpha coefficient of 0.644, falling short of the established criterion of 0.7. Although an alpha coefficient of 0.60–0.74 is still deemed clinically significant (Cicchetti, 1994), the scale's reliability remained within an acceptable range.

CFA revealed that the model fit met statistical standards. The convergent validity results indicated that all factors had a CR > 0.7. Regarding Factor 1, “willingness to engage in society,” and Factor 2, “dispelling anxiety about dementia,” the AVE values were slightly below 0.5 but above 0.38. The square roots of the above AVE values were greater than the correlation coefficients, demonstrating good discriminant validity (Shrestha, 2021). Therefore, convergent and discriminant validity were considered to be acceptable.

Criterion-related validity demonstrated correlations between the newly developed scale and existing measures from the PGC-MG, LSNS-6, and UCLA-Loneliness. The LSNS-6 correlations indicated that an increased social network was associated with greater dementia-related well-being. Conversely, the UCLA-Loneliness correlations showed that increased loneliness was associated with decreased dementia-related well-being. However, only the subscale “dispelling anxiety about dementia” did not exhibit a correlation with LSNS-6. This may be because network size alone cannot explain psychological factors (Kino et al., 2023), as isolation does not necessarily affect physical or mental health. Based on these results, we consider that criterion-related validity is confirmed.

## 6 Limitations and future issues

This study examined the reliability and validity of the DASFWB. However, it has several limitations. First, participants were aged 65 years and older. Stigma toward individuals with dementia is a pervasive issue that needs to be addressed regardless of age. Therefore, future research should expand the age range of participants and verify the reliability and validity of the scale. Second, the survey was conducted in a single, highly aged local municipality, limiting the generalizability of the results. Consequently, extending the survey to regions with different lifestyles is necessary to further assess the accuracy of the scale. Third, the survey targeted individuals without dementia. To build an inclusive society, assessing attitudes toward people with and without dementia is essential. To this end, we believe it is necessary to consider a scale that includes the cooperation of people with dementia.

## 7 Conclusion

The DASFWB was developed based on content validity, surface validity, item analysis, EFA, CFA, and criterion-related validity. The DASFWB comprised 12 items with three factors “willingness to engage in society,” “dispelling anxiety about dementia,” and “actions to take to cope with dementia” and its reliability and validity were verified.

## Data Availability

The original contributions presented in the study are included in the article/supplementary material, further inquiries can be directed to the corresponding author.
